# Ab initio MO study on direct production of H_2_O, N_2_O and CO_3_ from the respective CH_2_OO “Bee-sting-like” attack at H_2_, N_2_ and CO_2_

**DOI:** 10.1007/s00894-024-06065-1

**Published:** 2024-07-18

**Authors:** Hue-Phuong Trac, Ming-Chang Lin

**Affiliations:** 1 Center for Emergent Functional Matter Science, National Yang Ming Chiao Tung University, Hsinchu, Taiwan; 2 Department of Applied Chemistry, National Yang Ming Chiao Tung University, Hsinchu, Taiwan

**Keywords:** Ab initio MO calculation, CH_2_OO + H_2_, CH_2_OO + N_2_, CH_2_OO + CO_2_, Heat of formation of CH_2_OO

## Abstract

**Context:**

We have computationally elucidated the mechanism for formation of H_2_O, N_2_O and CO_3_ from the reactions of CH_2_OO with H_2_, N_2_ and CO_2_, respectively, by the direct attack of the terminal O atom of CH_2_OO. This unique mechanism, which is characteristically “bee-sting-like” in nature, was found to be closely parallel to their reactions with the O(^1^D) atom. Reactions with H_2_ and CO_2_ take place by side-on attack, while the N_2_ reaction occurs by end-on attack with predicted barriers, 19.4, 13.1 and 25.3 kcal.mol^−1^, respectively. The CO_2_ reaction with CH_2_OO was found to occur by producing the C_2v_ CO_3_, O = C < (O)O, instead of its D_3h_ conformer, essentially similar to the O(^1^D) + CO_2_ reaction. The rate constants for the three reactions have been computed by the transition state theory (TST) based on the predicted potential energy profiles. We have also utilized the isodesmic nature of the dative bond exchange in the N_2_ reaction, CH_2_O*** → ***O + N_2_ = CH_2_O + N_2_*** → ***O, to estimate the heat of the formation of CH_2_OO. Based on the heat of reaction computed at the highest level of theory employed, we obtained Δ_f_H^o^_0_ (CH_2_OO) = 27.5 kcal.mol^−1^; the value agrees with the recent results within ± 1 kcal.mol^−1^.

**Methods:**

All calculations were performed using Gaussian 16 software. Geometry, frequency, and IRC analysis calculations were conducted at the M06-2X/aug-cc-pVTZ level of theory. The heats of reaction have been evaluated at the highest level, CCSD(T)/CBS(T,Q,5)//M06-2x/aug-cc-pvTz.

**Supplementary Information:**

The online version contains supplementary material available at 10.1007/s00894-024-06065-1.

## Introduction

There have been voluminous publications since Welz et al. discovered in 2012 a convenient and clean method of producing the simplest Criegee intermediate (CI), CH_2_OO, by the CH_2_I + O_2_ → CH_2_OO + I reaction[1], which allowed experimentalists to monitor its kinetics cleanly. The kinetics and mechanisms of reactions of various CIs have been reviewed by many[2, 3]. Osborn and Taatjes[2] reviewed the physical chemistry of CIs in general in the gas phase. Vereecken and Francisco[3] discussed CIs’ complicated roles in the chemistry of troposphere. More recently, Chhantyal-Pun et al. detailed the production, detection and reactivity of various CIs[4].

Mechanistically, most of the reported reactions of atmospheric interest centered on bimolecular processes occurring via association complexes to be followed by fragmentation of these complexes, including the very fast head-to-tail self-reaction of the CH_2_OO zwitter-ionic species[5]. Truhlar and coworkers[6, 7] have recently carried out high-level computational studies on several atmospherically important CI reactions with a comprehensive listing of references for various pollutants, and H_2_O and its dimers, among many others. They put forth a triple-level strategy for prediction of atmospheric kinetics by employing a near complete-basis limit couple cluster theory with a new hybrid meta density functionals (MO6CR) optimized for CIs and a systematic treatment for the complex-forming kinetics [7]. Kumar and Francisco[8] theoretically examined the activation of X–H bonds by CIs for a series of molecules including H_2_, CH_4_, CH_x_F_4-x_ (x = 1–3) and SiH_4_, all taking place by the complex-forming mechanism. We will therefore not rehash these well-reported complex-forming bimolecular reactions in the present study.

In one of our computational studies on the thermal unimolecular decay of CH_2_OO[9], we postulated that the extension of the O–O bond following thermal excitation, the terminal O atom exhibited the O(^1^D)-atom character and was capable of inserting into one of the C-H bonds to form highly excited HCOOH with more than 110 kcal.mol^−1^ exothermicity, which enthalpically helped drive the formation and fragmentation of HCOOH to give various products, H_2_ + CO_2_, H_2_O + CO, among others[9]. A similar intramolecular insertion reaction was also illustrated for CH_3_HOO.

In the present study, we specifically investigated by a high-level quantum chemical calculation the O(^1^D)-atom character of the terminal O atom in bimolecular processes using the reactions of CH_2_OO with H_2_, N_2_ and CO_2_ as examples. Significantly, we discovered that H_2_O, N_2_O and CO_3_ could be formed *directly* from the respective reaction by a *Bee-sting-like (BSL) mechanism* without the need for very high activation energies. In nature, it is well-known that when a bee stings a person, its stinger detaches from its body and leaves inside the skin of the victim. To our knowledge, this type of unique bimolecular reaction mechanism involving two molecules has not been reported previously and is illustrated for the first time in this study. Parenthetically, we should mention that aside from the mechanistic elucidation of the BSL processes, we have also utilized the enthalpic change associated with the dative bond exchange, from CH_2_O*** → ***O to N_2_ giving CH_2_O + N_2_*** → ***O in the CH_2_OO + N_2_ reaction, to reliably evaluate the enthalpy of CH_2_OO formation. The results of this computational study are reported herein.

## Computational details

The mechanisms for the reactions of CH_2_OO with H_2_, N_2_ and CO_2_ have been studied by quantum-chemical calculations. The geometries of the reactants, products, and transition states were optimized by the M06-2X method[10] with the aug-cc-pVTZ basis set[11, 12] using the Gaussian 16 software package [13]. The optimized geometries at the M06-2X/aug-cc-pVTZ level were used to establish the potential energy surfaces (PESs) of the 3 reaction systems employing the coupled-cluster level of molecular orbital theory, CCSD(T), incorporating all the single and double excitations plus perturbative corrections for the triple excitations[14]. The zero-point energy (ZPE) corrections to the relative energies were also made at the M06-2X/aug-cc-pVTZ level. For the heats of reaction, we have also extrapolated the energies to the complete basis set (CBS) limit[15], based on the M06-2x/aug-cc-pvTz optimized geometries to obtain the CCSD(T)/CBS limit energies. The CBS energies were estimated using a three-point extrapolation scheme based on the CCSD(T) method, with the aug-pVXZ (X = T, Q, and 5) basis sets of Dunning[11].

Calculations of the kinetics for the direct exchange reactions, the conventional transition state theory (TST)[16] was employed by using the CHEMRATE code of NIST [17]. Tunneling corrections based on Eckart’s model have been made for H-transfer reactions[18]. The predicted reaction rates were obtained using the frequencies without scaling. All molecular parameters of the TSs, reactants, and products are listed in Table S1 of the SI section.

## Results and discussions

### CH_2_OO + H_2_

**Fig. 1:**
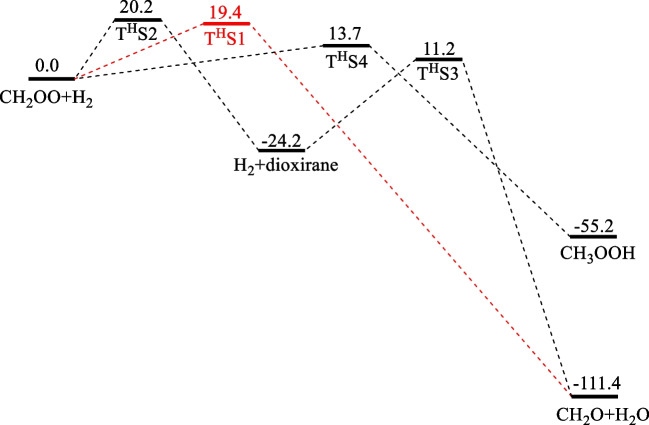
The potential energy surface for the CH_2_OO + H_2_ reaction by the direct terminal O-insertion step giving H_2_O via T^H^S1 computed at the CCSD(T)/CBS//M06-2X/aug-cc-pVTZ level (energies are in kcal.mol.^−1^)

Figure 1 presents the potential energy profile of the CH_2_OO + H_2_ reaction including the direct terminal O-insertion step giving H_2_O via T^H^S1 and those occurring by ring-TSs found in this work and in the work by Kumar and Francisco[8]. Our calculations were performed at the CCSD(T)/CBS//M06-2X/aug-cc-pVTZ level as aforementioned. For comparison, we have listed in Table 1 all the TS values and those of Kumar and Francisco’s reaction channels predicted at the CCSD(T)/aug-cc-pVTZ//M06-2X/aug-cc-pVTZ level. For the lowest energy barrier at T^H^S4, our result 13.7 kcal.mol^−1^ agrees closely with theirs, 13.4 kcal.mol^−1^; the CH_3_OOH product thus formed was predicted to lie 55.2 kcal.mol^−1^ below the reactants, which also agrees excellently with their value of 55.4 kcal.mol^−1^. We found another interesting reaction channel occurring by a ring TS, initiated by the H_2_-catalyzed isomerization CH_2_OO → H_2_C < (O)O (dioxirane) via T^H^S2 with a barrier of 20.2 kcal.mol^−1^. The dioxirane isomer further reacts with H_2_ via T^H^S3 (11.4 kcal.mol^−1^) to produce CH_2_O + H_2_O; this reaction path has been confirmed by an IRC analysis. Significantly, the insertion barrier at T^H^S1, 19.4 kcal.mol^−1^, lies between T^H^S2 (20.2 kcal.mol^−1^) and T^H^S4 (13.7 kcal.mol^−1^). The good agreement between our values evaluated at the CCSD(T)/CBS//M06-2X/aug-cc-pVTZ level with Kumar and Francisco’s result obtained at the CCSD(T)/aug-cc-pVTZ//M06-2X/aug-cc-pVTZ level for the barrier at T^H^S4 and the heat of reaction producing the CH_3_OOH product suggests that both methods are suitable for the CH_2_OO + H_2_ system, and the result for the direct BSL reaction via T^H^S1 is expected to be reasonable. The IRC analysis carried out for the reaction producing H_2_O by direct insertion is shown in Fig. S1.

**Table 1:** All TSs and intermediates of the reaction of CH_2_OO + H_2_ were computed at the CCSD(T)/CBS//M06-2X/aug-cc-pVTZ level

Species	This work	Reference
CH_2_OO + H_2_	0.0	
T^H^S1	19.4	
T^H^S2	20.2	
T^H^S4	13.7	13.4[8]
CH_3_OOH	-55.2	-55.4[8]
H_2_ + dioxirane	-24.2	
CH_2_O + H_2_O	-111.4	

### CH_2_OO + N_2_

**Fig. 2:**
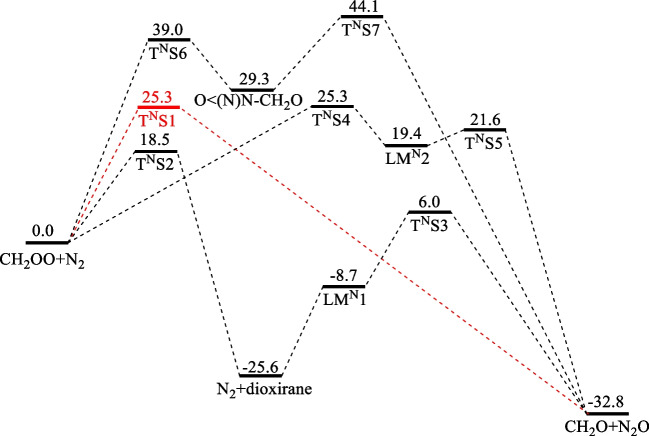
The potential energy surface for the CH_2_OO + N_2_ reaction by the direct terminal O-insertion step giving N_2_O via T^N^S1 computed at the CCSD(T)/CBS//M06-2X/aug-cc-pVTZ level (energies are in kcal.mol.^−1^)

To our knowledge, there has been no prior reported study on N_2_ reactions with CIs, CH_2_OO particularly. Our predicted PES of the CH_2_OO + N_2_ reaction at the CCSD(T)/CBS//M06-2X/aug-cc-pVTZ level is depicted in Fig. 2 with the energies summarized in Table 2. The barrier for the direct reaction of the terminal O atom with N_2_ at T^N^S1, 25.3 kcal.mol^−1^, is higher than that of the analogous H_2_ reaction, 19.4 kcal.mol^−1^, at T^H^S1 by about 6 kcal.mol^−1^. The IRC analysis of the reaction channel is shown in Fig. S2. There are several indirect redox paths for the ultimate transfer of the terminal O atom from CH_2_OO to N_2_. The 1st indirect path takes place via T^N^S4 (25.3 kcal.mol^−1^), forming a 5-member ring cyc-H_2_C-O–O-N–N- (LM^N^2); T^N^S4 has the same barrier as that of the direct channel. The ring intermediate, lying about 6 kcal.mol^−1^ below T^N^S4, readily fragments to CH_2_O + N_2_O with a small barrier of about 2 kcal.mol^−1^. The 2nd indirect path via T^N^S2 is mechanistically interesting and akin to the step in the H_2_ reaction via T^H^S2 (20.2 kcal.mol^−1^) through collision-induced CH_2_OO → CH_2_ < (O)O (dioxirane) isomerization with a slightly smaller barrier, 18.5 kcal.mol^−1^. Following the isomerization, the collision pair forms a stable molecular complex which further associates to yield a 5-member ring intermediate, cyc-CH_2_-O-N–N-O- (LM^N^1) with a binding energy 8.7 kcal.mol^−1^ below the reactants, CH_2_OO + N_2_. The intermediate readily fragments to CH_2_O + N_2_O, lying 32.8 kcal.mol^−1^ below the reactants. The highest barrier reaction also occurs, interestingly, by direct terminal O-atom attack of N_2_, side-on instead of end-on as in T^N^S1, forming CH_2_O + O < (N)N. The ring product O < (N)N can undergo isomerization via T^N^S7 to N_2_O with a 14.8 kcal.mol^−1^ barrier, accompanying with the overall exothermicity of 32.8 kcal.mol^−1^.

**Table 2:** All TSs and intermediates of the reaction of CH_2_OO + N_2_ were computed at the CCSD(T)/CBS//M06-2X/aug-cc-pVTZ level

Species	This work
CH_2_OO + N_2_	0.0
T^N^S1	25.3
T^N^S2	18.5
T^N^S3	6.0
T^N^S4	25.3
T^N^S5	21.6
T^N^S6	39.0
T^N^S7	44.1
LM^N^1	-8.7
LM^N^2	19.4
O < (N)N-CH_2_O	29.3
N_2_ + dioxirane	-25.6
CH_2_O + N_2_O	-32.8

Mechanistically, the overall reaction CH_2_OO + N_2_ → CH_2_O + N_2_O involves the exchange of the dative bond (→ O) from CH_2_OO to N_2_. This important property will be utilized in Sect. 2.4 for estimation of the enthalpy of formation of CH_2_OO.

### CH_2_OO + CO_2_

**Fig. 3:**
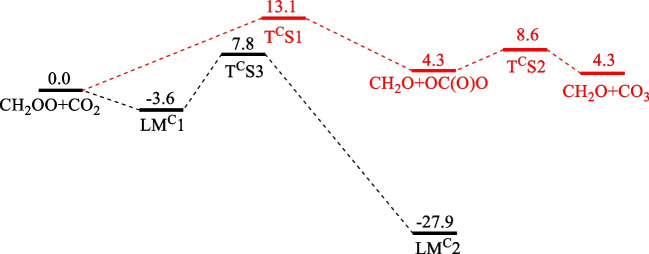
The potential energy surface for the CH_2_OO + CO_2_ reaction by the direct terminal O-insertion step giving CO_3_ via T^C^S1 and T^C^S2 computed at the CCSD(T)/CBS//M06-2X/aug-cc-pVTZ level (energies are in kcal.mol.^−1^)

Figure 3 shows the potential energy profile of the CH_2_OO + CO_2_ reaction forming CO_3_ by T^C^S1 and the lower energy product channels taking place by initial ring complex formation as reported by Kumar and Francisco[19] and Lin et al.[20]. Our values for the complex forming path obtained at the CCSD(T)/CBS//M06-2X/aug-cc-pVTZ level are listed in Table 3 for comparison with the results of Kumar and Francisco predicted at the CCSD(T)/aug-cc-pVTZ//M06-2X/aug-cc-pVTZ level[19] and the experimentally validated values of Lin et al. computed at the QCISD(T)/CBS//B3LYP/6–311 + G(2d,2p) level[20]. The barrier at T^C^S3, 7.8 kcal.mol^−1^, the energies for the pre-reaction (LM^C^1) and postreaction (LM^C^2) intermediates, -3.6 kcal.mol^−1^ and -27.9 kcal.mol^−1^, respectively, all agree closely their values as presented in Table 3. The collision-induced isomerization of CH_2_OO to CH_2_ < (O)O, akin to those occurring via T^H^S2 and T^N^S2 mentioned above, was found to have an energy barrier of 17 kcal.mol^−1^ above the reactants at the M06-2X/aug-cc-pVTZ level; no further calculations were performed because of the high barrier.

**Table 3:** All TSs and intermediates of the reaction of CH_2_OO + CO_2_ were computed at the CCSD(T)/CBS//M06-2X/aug-cc-pVTZ level

Species	This work	Reference
CH_2_OO + CO_2_	0.0	
T^C^S1	13.1	
T^C^S2	8.6	
T^C^S3	7.8	6.9[19], 8.1[20]
LM^C^1	-3.6	-4.1[19], -3.9[20]
LM^C^2	-27.9	-29.0[19], -27.4[20]
CH_2_O + CO_3_	4.3	

For the reaction channel of interest to this work, the direct formation of CO_3_ by the terminal O-atom attack at CO_2_, the mechanism turns out to be quite interesting. The initial CO_3_ product was found to have the C_2v_ structure, O = C < (O)O, formed by side-on attack; O = C < (O)O isomerizes readily to its D_3h_ conformer with a small barrier of 4.3 kcal.mol^−1^. The IRC analysis of the reaction channel is shown in Fig. S3. This mechanism turns out to be similar to that of the O(^1^D) + CO_2_ reaction predicted by Mebel and coworkers[21], in which the C_2v_ CO_3_ was found to be produced directly. The C_2v_ to D_3h_ CO_3_ isomerization barrier was reported to be 4.4 kcal.mol^−1^ at the MRCI + Q(16,13)/6–311 + G(3df) level of theory, in very good agreement with our value, 4.3 kcal.mol^−1^, computed at the CCSD(T)/CBS//M06-2X/aug-cc-pVTZ level as aforementioned. Parenthetically, we should mention that the singlet CO_2_-oxide, O = CO*** → ***O, was found to be theoretically unstable.

## Heat of Formation of CH_2_OO

The isodesmic nature of the dative bond exchange in the N_2_ reaction,

$${\text{CH}}_{2}\text{O}\to \text{O }+ {\text{N}}_{2} = {\text{CH}}_{2}\text{O }+ {\text{N}}_{2}\to \text{O}$$

as alluded above, is ideal for estimation of the heat of formation of CH_2_OO using the reliably predicted heat of the reaction (Δ_r_H^o^) and the experimentally well-established heats of formation of CH_2_O and N_2_O. The energy balance of the reaction gives rise to: Δ_f_H^o^ (CH_2_OO) = Δ_r_H^o^ + Δ_f_H^o^ (CH_2_O) + Δ_f_H^o^ (N_2_O) at 0 K. Based on the values of Δ_r_H^o^ predicted at the 3 different levels of theory, we obtained: I, at the CCSD(T)/CBS(D,T,Q)//M06-2x/aug-cc-pvTz level, 28.1 kcal.mol^−1^; II, at the CCSD(T)/CBS(T,Q,5)//M06-2x/aug-cc-pvTz level, 27.5 kcal.mol^−1^; and III, at the M06-2x/CBS(D,T,Q)//M06-2x/aug-cc-pvTz level, 29.3 kcal.mol^−1^; the 3 closely banded values: 28.1, 27.5 and 29.3 kcal.mol^−1^ are presented in Table 4. Our highest level result, 27.5 kcal.mol^−1^, lies closely within the recently predicted value of Nguyen et al.[22], 28.1 kcal.mol^−1^ acquired at the CCSD(T)/CBS(D,T,Q)//CCSD(T)/aug-cc-pvTz level, and the most recent high-level prediction, 26.5 kcal.mol^−1^, determined at the CCSDTQ/CBS(D,T,Q,5,6) + Δ//CCSD(T)/ANO2 level of theory [23]. It is worth mentioning that above results are also in reasonable agreement with the earlier value of 27.0 at 298 K (or 28.7 kcal.mol^−1^ at 0 K) reported by Cremer and coworkers[24], obtained by the CCSD(T)/[4s3p2d1f/3s2p1d] calculation.

**Table 4:** Heat of Formation of CH_2_OO predicted by different authors (in kcal.mol^−1^ at 0 K)

	This work ^a^			Reference
	I	II	III	
Δ_f_H^o^_0_	28.1	27.5	29.3	28.1^b^, 26.5^c^, 28.7^d^

^**a**^I, CCSD(T)/CBS(D,T,Q)//M06-2x/aug-cc-pvTz; II, CCSD(T)/CBS(T,Q,5)//M06-2x/aug-cc-pvTz; and III, M06-2x/CBS(D,T,Q)//M06-2x/aug-cc-pvTz

^**b**^CCSD(T)/CBS(D,T,Q)//CCSD(T)/aug-cc-pvTz (ref. [22])

^**c**^CCSDTQ/CBS(D,T,Q,5,6) + Δ//CCSD(T)/ANO2 (ref [23])

^d^CCSD(T)/[4 s 3p 2d 1f/3s2p1d] (ref [24])

## Conclusions

In this study, we have explored the reactivity of the terminal O atom of CH_2_O*** → ***O toward H_2_, N_2_ and CO_2_ directly producing H_2_O, N_2_O and CO_3_, respectively, by the bee-sting-like mechanism without going through formation of complexes. This unique reaction mechanism involving two singlet molecular species has not been reported before. The reaction paths have been clearly illustrated by IRC analyses (see Figs. S1, S2 and S3). Concurrently, we also examined most, if not all, of the processes taking place by indirect complexing mechanisms. Comparison of the energetics involved in many of these processes previously investigated by several authors with our values obtained from calculations at the CCSD(T)/CBS(D,T,Q)//M06-2x/aug-cc-pvTz level of theory agrees very closely.

**Fig. 4:**
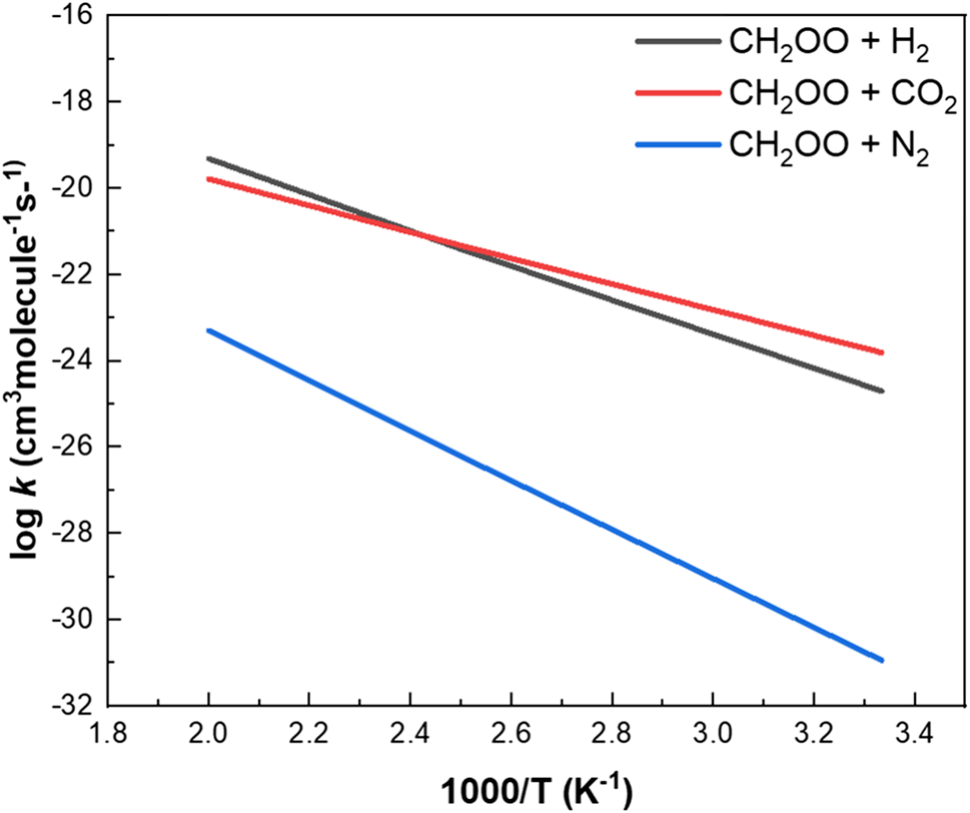
Predicted rate constants for CH_2_OO reactions with H_2_, N_2_ and CO_2_ by direct “bee-sting-like” attacks of the terminal O-atom producing H_2_O, N_2_O and CO_3_, respectively

For the direct reactions, H_2_O was found to be formed by insertion of the terminal O atom into H_2_ side-on after the extension of the O*** → ***O bond at transition state T^H^S1 with the barrier energy of 19.4 kcal.mol^−1^, while N_2_O and CO_3_ were formed by the attack of the terminal O atom, end-on and side-on at T^N^S1 and T^C^S1, respectively, with largely different barriers of 25.3 and 13.1 kcal.mol^−1^. Significantly, the predicted rate constants at 300 K (see Fig. 4) were found to correlate qualitatively with the rate constants for the O(^1^D) atom reactions with H_2_, N_2_ and CO_2_ at 300 K. The similarity was even more vividly illustrated by the mechanism of the CH_2_OO + CO_2_ reaction, as shown in Fig. 3, that the C_2v_ CO_3_, O = C < (O)O, instead of its D_3h_ conformer, was initially formed in the reaction, akin to that occurs in the O(^1^D) + CO_2_ reaction reported by Mebel and coworkers[21]. The O(^1^D)-like character of the terminal O atom exhibited in these bimolecular reactions indirectly corroborates our previous study on the unimolecular decay of CH_2_OO by intramolecular insertion of the terminal O atom into one of its C-H bonds forming HCOOH very exothermically[9].

We have utilized the isodesmic characteristics of the dative bond exchange in the N_2_ reaction,

$${\text{CH}}_{2}\text{O}\to \text{O }+ {\text{N}}_{2} = {\text{CH}}_{2}\text{O }+ {\text{N}}_{2}\to \text{O},$$

for estimation of the heat of formation of CH_2_OO. We believe that this is perhaps one of the most reliable schemes for evaluation of the heat of CH_2_OO formation theoretically because of the well-established thermochemistry of CH_2_O and N_2_O, and the potential computational-error cancellation from each side of the reaction as well. Based on the heat of the above reaction evaluated with our highest level of theory, CCSD(T)/CBS(T,Q,5)//M06-2x/aug-cc-pvTz, the heat of formation of CH_2_OO was predicted to be 27.5 kcal.mol^−1^. The value agrees with the best recent estimates[22–24] approximately within ± 1 kcal.mol^−1^.

### Supplementary Information

Below is the link to the electronic supplementary material.

Supplementary file1 (DOCX 4450 KB)

## Data Availability

No datasets were generated or analysed during the current study.
