# Supplementary material for: Ab initio MO study on direct production of H2O, N2O and CO3 from the respective CH2OO “Bee-sting-like” attack at H2, N2 and CO2
Source: J Mol Model. 2024 Jul 18;30(8):272. doi: 10.1007/s00894-024-06065-1 (PMC11258077; doi:10.1007/s00894-024-06065-1)
Supplement: Supplementary file 1 — Supplementary file1 (DOCX 4450 KB) [file 894_2024_6065_MOESM1_ESM.docx]

**Supporting Information**

***Ab Initio* MO Study on Direct Production of H_2_O, N_2_O and CO_3_ from the Respective CH_2_OO “Bee-sting-like” Attack at H_2_, N_2_ and CO_2_**

Hue-Phuong Trac^1,2^ and Ming-Chang Lin^1,2*^

^1^Center for Emergent Functional Matter Science, National Yang Ming Chiao Tung University, Hsinchu, Taiwan.

^2^Department of Applied Chemistry, National Yang Ming Chiao Tung University, Hsinchu, Taiwan.

***Corresponding authors:** [chemmcl@emory.edu](mailto:chemmcl@emory.edu)

**Fig. S1.** The IRC analysis of the reaction CH_2_OO + H_2_ producing H_2_O + CH_2_O.

**Fig. S2** The IRC analysis of the reaction CH_2_OO + N_2_ producing N_2_O + CH_2_O.

**Fig. S3.** The IRC analysis of the reaction CH_2_OO + CO_2_ producing CO_3_ + CH_2_O.

**Fig. S4.** Molecular structures of the reactants, key intermediates transition states, and products involved in the CH_2_OO + H_2_ reaction optimized at the M06-2X/aug-cc-pVTZ level. Bond angles and bond lengths are in degree (^o^) and angstroms (Å), respectively.

**Fig. S5.** Molecular structures of the reactants, key intermediates transition states, and products involved in the CH_2_OO + N_2_ reaction optimized at the M06-2X/aug-cc-pVTZ level. Bond angles and bond lengths are in degree (^o^) and angstroms (Å), respectively.

**Fig. S6.** Molecular structures of the reactants, key intermediates transition states, and products involved in the CH_2_OO + CO_2_ reaction optimized at the M06-2X/aug-cc-pVTZ level. Bond angles and bond lengths are in degree (^o^) and angstroms (Å), respectively.

**Table S1**. Vibrational frequencies and moments of inertia (I_A_, I_B_, I_C_) of the species in the reactions of CH_2_OO with H_2_, N_2_, and CO_2_ computed at the M06-2X/aug-cc-pVTZ level.

| Species or  Transition states | Moments of inertia I_A_, I_B_, I_C_ (a.u) | Vibrational Frequencies (cm^-1^) |
| --- | --- | --- |
| CH_2_OO | 22.3, 142.3, 164.6 | 538, 698, 929, 1023, 1261, 1433, 1630, 3140, 3291 |
| H_2_ | 0.0, 1.0, 1.0 | 4467 |
| T^H^S1 | 33.2, 218.5, 251.7 | 28, 285, 384, 385, 606, 644, 800, 1201, 1249, 1479, 1813, 3042, 3157, 3872, 1120*i* |
| T^H^S2 | 46.3, 145.2, 172.4 | 561, 713, 804, 893, 1013, 1127, 1191, 1237, 1403, 1422, 1561, 2636, 3148, 3278, 1004*i* |
| T^H^S3 | 64.1, 243.3, 307.4 | 108, 171, 220, 259, 333, 365, 469, 1218, 1238, 1484, 1854, 2958, 3049, 4171, 891*i* |
| T^H^S4 | 91.1, 143.8, 169.7 | 83, 161, 239, 330, 450, 790, 813, 1102, 1229, 1452, 1616, 3080, 3228, 4388, 836*i* |
| Dioxirane | 62.5, 67.5, 117.6 | 910, 954, 1050, 1186, 1272, 1375, 1560, 3106, 3206 |
| CH_3_OOH | 40.9, 169.1, 193.5 | 222, 256, 468, 955, 1115, 1183, 1228, 1402, 1460, 1479, 1518, 3049, 3120, 3154, 3830 |
| H_2_O | 2.2, 4.2, 6.4 | 1620, 3869, 3972 |
| CH_2_O | 6.3, 45.7, 52.1 | 1213, 1273, 1540, 1869, 2946, 3016 |
| N_2_ | 0.0, 29.5, 29.5 | 2523 |
| T^N^S1 | 40.2, 1010.4, 1050.7 | 61, 63, 217, 320, 350, 502, 517, 1210, 1255, 1493, 1831, 2469, 3020, 3126, 715*i* |
| T^N^S2 | 161.1, 709.5, 795.4 | 37, 60, 63, 80, 115, 791, 809, 1108, 1230, 1453, 1617, 2527, 3083, 3228, 830*i* |
| T^N^S3 | 184.6, 253.6, 425.9 | 110, 435, 593, 643, 796, 967, 1171, 1227, 1300, 1400, 1578, 2006, 3038, 3120, 607*i* |
| T^N^S4 | 184.3, 256.0, 405.6 | 430, 445, 471, 678, 727, 1003, 1048, 1229, 1249, 1310, 1511, 2065, 3117, 3239, 436*i* |
| T^N^S5 | 183.2, 238.0, 409.1 | 193, 524, 616, 713, 871, 1020, 1186, 1225, 1295, 1330, 1544, 1861, 3035, 3112, 642*i* |
| T^N^S6 | 74.6, 836.9, 850.4 | 40, 138, 175, 273, 314, 356, 437, 1219, 1263, 1512, 1848, 2275, 2990, 3084, 550*i* |
| T^N^S7 | 171.1, 639.6, 761.3 | 39, 54, 87, 98, 131, 162, 584, 1228, 1280, 1533, 1854, 2159, 2959, 3048, 752*i* |
| LM^N^1 | 184.2, 196.1, 366.7 | 103, 609, 764, 824, 966, 1013, 1056, 1113, 1170, 1197, 1417, 1562, 1697, 3061, 3148 |
| LM^N^2 | 187.2, 201.2, 373.4 | 138, 567, 770, 789, 906, 933, 1023, 1098, 1102, 1215, 1355, 1522, 1737, 3053, 3149 |
| O<(N)N-CH_2_O | 140.6, 664.2, 737.3 | 33, 43, 82, 96, 112, 151, 560, 798, 1224, 1277, 1535, 1860, 2001, 2958, 3039 |
| N_2_O | 0.0, 140.4, 140.4 | 652, 652, 1359, 2423 |
| CO_2_ | 0.0, 152.5, 152.5 | 694, 694, 1411, 2443 |
| T^C^S1 | 214.0, 635.2, 813.1 | 101, 191, 252, 341, 467, 481, 681, 754, 848, 1198, 1250, 1298, 1498, 1775, 1968, 3056, 3176, 534*i* |
| T^C^S2 | 236.4, 850.6, 1014.7 | 41, 97, 116, 142, 183, 669, 698, 782, 814, 1120, 1234, 1406, 1457, 1616, 2436, 3080, 3240, 834*i* |
| T^C^S3 | 215.3, 566.1, 742.9 | 133, 250, 403, 445, 589, 688, 741, 780, 945, 1123, 1244, 1307, 1447, 1640, 2134, 3155, 3295, 381*i* |
| LM^C^1 | 240.1, 804.4, 1008.6 | 55, 118, 131, 170, 209, 539, 660, 698, 698, 914, 1057, 1262, 1402, 1442, 1650, 2431, 3144, 3290 |
| LM^C^2 | 202.1, 456.1, 633.4 | 202, 296, 528, 721, 768, 781, 945, 990, 1080, 1138, 1157, 1212, 1261, 1441, 1539, 1973, 3074, 3183 |
| CO_3_ | 67.3, 195.3, 262.6 | 585, 679, 805, 962, 1189, 2143 |
